# Barriers and Strategies in Guideline Implementation—A Scoping Review

**DOI:** 10.3390/healthcare4030036

**Published:** 2016-06-29

**Authors:** Florian Fischer, Kerstin Lange, Kristina Klose, Wolfgang Greiner, Alexander Kraemer

**Affiliations:** 1 Department of Public Health Medicine, School of Public Health, Bielefeld University, 33615 Bielefeld, Germany; kerstin.lange@uni-bielefeld.de (K.L.); alexander.kraemer@uni-bielefeld.de (A.K.); 2 Department of Health Care Management, School of Public Health, Bielefeld University, 33615 Bielefeld, Germany; kristina.klose@uni-bielefeld.de (K.K.); wolfgang.greiner@uni-bielefeld.de (W.G.)

**Keywords:** guideline implementation, scoping review, barrier, strategy

## Abstract

Research indicates that clinical guidelines are often not applied. The success of their implementation depends on the consideration of a variety of barriers and the use of adequate strategies to overcome them. Therefore, this scoping review aims to describe and categorize the most important barriers to guideline implementation. Furthermore, it provides an overview of different kinds of suitable strategies that are tailored to overcome these barriers. The search algorithm led to the identification of 1659 articles in PubMed. Overall, 69 articles were included in the data synthesis. The content of these articles was analysed by using a qualitative synthesis approach, to extract the most important information on barriers and strategies. The barriers to guideline implementation can be differentiated into personal factors, guideline-related factors, and external factors. The scoping review revealed the following aspects as central elements of successful strategies for guideline implementation: dissemination, education and training, social interaction, decision support systems and standing orders. Available evidence indicates that a structured implementation can improve adherence to guidelines. Therefore, the barriers to guideline implementation and adherence need to be analysed in advance so that strategies that are tailored to the specific setting and target groups can be developed.

## 1. Introduction

Clinical practice guidelines (hereafter referred to as guidelines) can be defined as “systematically developed statements to assist practitioners’ decisions about appropriate health care for specific clinical circumstances” [1]. Therefore, guidelines are considered to decrease the gap between research and current practice and, thus, to reduce inappropriate variability in practice [2]. Guidelines are valuable tools in situations where the scientific evidence is sparse, where multiple therapies are available, or where uncertainty in terms of treatment options exists. The development and implementation of guidelines is intended to organize and provide the best available evidence to support clinical decision making in order to improve quality of care, patient outcomes and cost effectiveness [3,4]. The criteria and prerequisites for developing guidelines are: a highly prevalent disease or frequently used medical procedure, high associated costs and current variations in practice. They are particularly important for diseases leading to premature mortality, avoidable morbidity or negative effects on health-related quality of life. Furthermore, the evidence should indicate that medical care can make a difference to outcomes [5].

Guidelines may be used to translate medical research and expert opinions into recommendations for the daily practical work of health professionals [6]. Despite the growing number of guidelines, their use in practice is frequently reported as being unpredictable, often slow and complex [6,7,8]. Research indicates that guidelines are often not applied. This does not include specific and justified actions in single cases to treat in ways other than those described in the guidelines for the sake of patients, but non-adherence to guidelines may lead to unnecessary diagnostics and suboptimal or even inadequate treatment [9]. It is estimated that about 30%–40% of patients receive treatment that is not based on scientific evidence, and 20%–25% receive treatments that are either not needed or potentially harmful [6,10,11]. Therefore, more research on the implementation of guidelines is needed to promote the systematic translation of current research evidence into routine practice [12]. A successful introduction of guidelines involves the three steps of development, dissemination and implementation [13]. During the past few years, it has become obvious that the development of a guideline does not necessarily lead to changes in clinical practice. Therefore, the focus of management and research in this area has changed from the development to the implementation of guidelines. For example, the latest guidelines have a stronger focus on patient-relevant outcomes and patient involvement during their development, and contain summaries and patient versions to support the implementation process. Nevertheless, knowledge regarding appropriate strategies to implement guidelines remains sparse and no implementation strategy has been identified that is effective in all circumstances [14].

Implementation strategies have to be based on current knowledge about potentially effective interventions and upon an assessment of potential barriers to guideline adoption [10,15,16,17,18,19,20]. The identification of barriers may subsequently lead to the development of tailored implementation strategies. Nevertheless, evidence regarding the most suitable way to translate the identified barriers into tailored interventions is missing [20]. The implementation of guidelines is a complex process that is hampered by several barriers [21,22,23,24]. A systematic review by Cabana and colleagues of barriers to physician adherence to guidelines that included 76 studies identified a large number of different barriers. The authors developed a framework in which these barriers were classified into three main categories: barriers related to physicians’ knowledge (e.g., lack of awareness and lack of familiarity), barriers that affect physicians’ attitudes (e.g., lack of agreement and lack of motivation) and external barriers (e.g., patient-, guideline- and environment-related factors) [22].

The success of any implementation depends on the consideration of a variety of barriers and the use of adequate strategies to overcome them. Therefore, this study aims to describe and categorize the most important barriers to guideline implementation identified by a scoping review. Since the systematic literature review by Cabana and colleagues was published back in 1999, an update is needed which takes the current situation into account. Until now, studies have been divided between barriers and implementation strategies and have focused on only one aspect. To our knowledge, this is the first study to provide an overview of different kinds of suitable strategies that are directly related to the underlying barriers. This study should provide information on barriers and the strategies that are needed to promote the effective implementation and incorporation of guidelines into practice.

## 2. Materials and Methods

A scoping review was conducted in PubMed according to the procedure and requirements described in the Preferred Reporting Items for Systematic Reviews and Meta-Analyses (PRISMA) statement [25]. This type of review was chosen because the area of interest is complex. By conducting a scoping review, we aimed to comprehensively identify and describe both barriers to and strategies for guideline implementation. A filter was used to restrict the search to the English and German languages. All articles listed in PubMed and published up until the end of 2015 were considered. The evolved search algorithm focused on several search terms mentioned in the title and abstract. To narrow the search results, synonyms for “guideline” were combined with the search terms “implementation”, “barriers or strategies” and further search terms that indicate aspects of “compliance, acceptance, conformity, approval or adherence”. Therefore, the following search algorithm was used:

(guideline*[Title/Abstract] OR guidance*[Title/Abstract] OR clinical protocol*[Title/Abstract]) AND (strateg*[Title/Abstract] OR barrier*[Title/Abstract]) AND implement*[Title/Abstract] AND (compliance[Title/Abstract] OR accept*[Title/Abstract] OR conform*[Title/Abstract] OR approv*[Title/Abstract] OR adherence[Title/Abstract]) 

This search algorithm led to the identification of 1659 articles. After screening of title and abstract, 1512 of these articles were excluded because they did not fit the study’s objective. Therefore, 146 articles were considered in the screening of full texts. By means of this assessment, 82 were excluded for the following reasons:

- exclusively disease-specific information on barriers and/or strategies, which do not allow for generalizations (*n* = 51),
- no direct reference to barriers or strategies for guideline implementation (*n* = 38),
- no clinical guidelines (*n* = 9),
- no comparability (e.g., developing countries) (*n* = 7),
- study protocol (*n* = 2).

**Figure 1:**
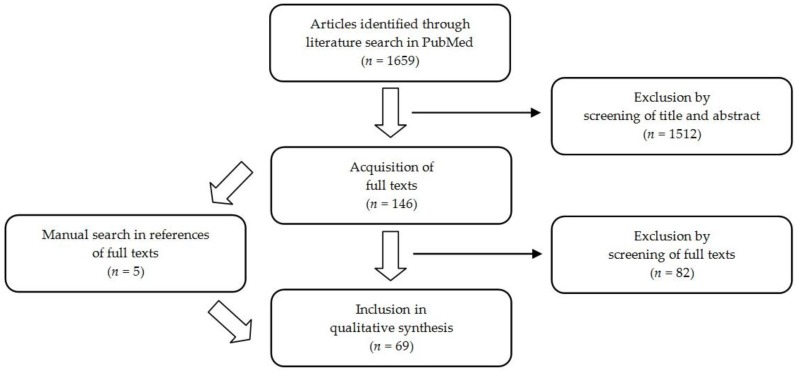
Flow diagram of the review.

The number of articles excluded for each criterion is given in brackets. The sum does not add up to 82 because some articles were excluded for multiple reasons. A manual search was conducted through the reference lists of all full texts, which led to the inclusion of five further articles. Finally, 69 articles were included in the qualitative analysis of the scoping review (see Figure 1).

The content of the articles was analysed by using a qualitative synthesis approach, in order to extract the most important information on barriers and strategies. Furthermore, during the process of analysis special attention was given to the link between barriers and adequate strategies.

Studies and reviews were included in the synthesis. If barriers and/or strategies were mentioned, they were included in the framework that we developed as a result of our study. For this reason, no quality appraisal tools were used.

## 3. Results

Overall, the 69 articles included in the synthesis were composed of 42 studies and 27 reviews or theoretical conceptual papers. Most articles described the situation regarding guideline implementation in a particular country, mainly the Netherlands (*n* = 15), the United States of America (*n* = 13), Australia (*n* = 7) and Germany (*n* = 6). In 47 articles, barriers or strategies regarding the implementation of disease-specific guidelines were described; 22 articles did not concentrate on a specific disease but described barriers and strategies in general. Overall, 32 articles include information on barriers and 49 describe strategies for guideline implementation. Some articles described both barriers and strategies (*n* = 12), although they were not always directly linked to each other. Further information on the articles included in the qualitative synthesis is given in Table 1.

### 3.1. Barriers and Strategies in Guideline Implementation

We have summarised the barriers, interventions and strategies involved in guideline implementation that were mentioned in the chosen articles in Table 2. Single interventions were separated from more complex strategies, which mainly consist of a combination of single interventions or provide a broader perspective on solutions to solve the problems created by barriers to guideline implementation. Only those aspects which are of general importance and not disease- or guideline-specific were included. We distinguished between personal factors, guideline-related factors and external factors, to highlight the different levels upon which strategies may focus.

The barriers mentioned under *personal factors* are divided into factors related to physicians’ knowledge and attitudes because these two factors are closely linked to any behavioural change and are prerequisites for it [22]. In terms of physicians’ knowledge, the main barriers to implementation and adherence are lack of awareness and lack of familiarity with the guideline and its recommendations [22,33]. When it comes to attitudes, the main barriers are considered to be: lack of agreement, self-efficacy, skills, outcome expectancy and motivation [22,33,49]. In this context, the derived strategies for guideline implementation mainly focus on dissemination strategies and educational aspects. In particular, active learning from experts as opinion leaders [14,23,28,71,85] and continuing education [20,28], e.g., by continuous medical education (CME) [82,86], were emphasized as useful tools for improving physicians’ knowledge. Regarding the improvement of physicians’ attitudes, (individualized) audit and feedback are considered to be effective strategies [32,36,43,49].

The main barriers connected with *guideline-related factors* are linked to the process of developing and establishing a guideline. In particular, evidence and the plausibility of recommendations are important factors during the development phase. Furthermore, the complexity [27,43,50], layout [57], accessibility [45] and applicability [53] have to be considered. Therefore, the guidelines should be as short and user-friendly as possible [86] to reduce complexity [27,43,50,53]. Checklists [84] and further tools, such as the inclusion of tablets, smartphones and mobiles as platforms for the dissemination of guidelines [62] and the implementation of decision support systems [30,57], were mentioned as suitable strategies to improve accessibility. The quality of a guideline, which is associated with its usage, may also depend upon the consideration of comorbidity and multimorbidity within its recommendations. Clear intervention goals must be set [23].

*External factors* may also confer several advantages during the implementation process of a guideline. Among the barriers related to external factors, organizational constraints are of major importance. Therefore, improvements in the organization of care are necessary, which may be promoted by the standardization of processes and procedures [87] and the development of protocols [57]. In addition, the care setting has to be considered during the development of a guideline [46,88] and links to quality management [54] may improve adherence. The barrier of lack of resources, such as time restrictions and heavy workload [35,55,68], can only be overcome by the provision of enough time to utilize the guidelines in practice [31,69], by establishing clear roles in terms of standing orders [31] and by providing financial incentives [42,54]. A multiprofessional collaboration with other healthcare professionals may foster implementation and adherence to guidelines [20,56]. The local context must also be considered to allow for the local adaptation of guidelines [23,50,61]. Furthermore, local consensus groups may change social norms and, therefore, improve guideline implementation [85].

### 3.2. Categorization of Implementation Strategies

The description of the barriers related to guideline implementation highlights the fact that hindrances to the adoption of and adherence to guidelines exist at different levels. Therefore, both the general and context-specific barriers have to be assessed and analysed to allow for the development of tailored implementation strategies [29,50,57]. Furthermore, the patient perspective needs to be recognized [36,41]. An overview of several barriers is provided in Table 2, but many more barriers related to the content of guidelines or the medical disciplines are conceivable and should be considered during a structured implementation process.

Strategies for guideline implementation can be broadly classified as workflow- or provider-focused. Workflow-focused strategies seek to minimize contextual barriers and promote changes that facilitate the adoption of guidelines (e.g., clinical reminders). Provider-focused strategies seek to minimize provider-level barriers and to create provider-level facilitators to guideline adherence. This can be achieved by the use of communication strategies to raise awareness about guidelines. These two types of strategy are complementary. Therefore, the effective implementation of guidelines requires a combination of workflow- and provider-focused strategies [42]. It follows that a multifaceted implementation with a balanced mix between the two types of strategy is more likely to lead to guideline adherence [23,42,86]. In addition, several strategies identified and categorized during the review process contain both workflow- and provider-focused interventions.

The review revealed the following aspects as central elements of successful strategies for guideline implementation. One major factor in the process is the aspect of *dissemination*. Therefore, the supply of educational materials (including written materials, didactic presentations and interactive conferences) is absolutely essential to raise awareness and increase familiarity and agreement with a guideline and its recommendations [30,43,46,54]. Furthermore, continuous efforts in the *education and training* of health professionals are needed. This may be done by educational meetings and educational outreach visits [30], audit and feedback [36,37,43,49], workshops [50,89] and small-group interactive postgraduate training sessions [50]. *Social interaction* is mentioned as a highly relevant factor in guideline implementation. This interaction may include educational outreach visits and marketing [32]. In this context, local opinion leaders are capable of improving evidence-based practice because they may enable guideline developers to meet with the key staff at each organization [28,71]. It must be highlighted that the role of an opinion leader is not equivalent to a physician champion. Opinion leaders are often the first to know about guidelines and to adopt them into their daily clinical practice. In contrast, physician champions might be opinion leaders, but instead of exerting an informal influence, they actively participate in designing and implementing improvement, e.g., by promoting guideline adherence [39]. Two further strategies are workflow focused. Firstly, *decision support systems* (manual or automated) and reminders will prompt health professionals to perform clinical actions according to the current state of evidence [30]. Secondly, *standing orders* and standardized documentation are strategies to facilitate guideline adherence [32].

## 4. Discussion

**Figure 2:**
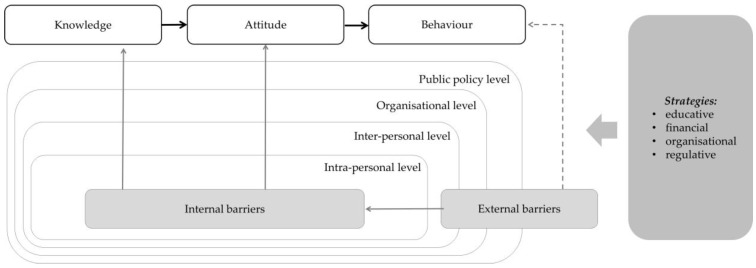
Knowledge-Attitude-Behaviour Framework: Barriers and strategies to guideline implementation.

The absorption of guideline recommendations into everyday practice requires changes in the attitudes and behaviour of health professionals and a certain adaptation of the structural environment [1,6,22,26]. Although behaviour can modify even in the absence of changes in knowledge and attitude, behavioural modifications based on such changes are more permanent [24]. All the barriers described in this article—both internal and external—may influence the knowledge, attitudes or behaviour of health professionals [33]. According to the “Knowledge-Attitude- Behaviour Framework”, physicians have to be aware of a guideline and need to have some knowledge of its content. Afterwards, knowledge influences attitudes, and attitudes affect practice behaviour [90]. Therefore, implementation strategies should be focused on the improvement of knowledge and attitudes in order to improve the uptake of guidelines in clinical practice [4] (see Figure 2).

In order for strategies that promote guideline implementation and adherence to be developed, the barriers have to be assessed and addressed. If interventions and implementation strategies are tailored to specific settings and target groups, behaviour change is more likely [16,29,88]. In this review, we have distinguished between personal factors, guideline-related factors and external factors. In further articles other differentiations were used. For example, a review by Baiardini et al. [24] distinguished between factors which are guideline related (e.g., level of evidence, plausibility, trialability), context dependent (e.g., social norms, organizational characteristics) or directly connected to implementation (e.g., communication and educational strategies). A qualitative study of perceived facilitators and barriers to guideline implementation in psychiatry highlighted the categories of organizational resources (e.g., staff, leadership and dissemination), healthcare professionals’ individual characteristics (knowledge, attitudes and beliefs) and the perception of guidelines and implementation strategies (credibility of content and awareness) [23]. Despite differences in the classification of barriers, successful implementation strategies need to address them all [4,10,22].

Since guidelines are not self-implementing, a step-by-step and planned introduction is needed [16]. Zwerver et al. [17] describe the intervention mapping process for guideline implementation, which was originally developed by Bartholomew et al. [18]. According to this plan, six basic steps must be performed during the process of guideline implementation. The first step is a needs assessment, which aims to identify the target group and stakeholders. In the second step, programme objectives are defined, in which the expected changes in behaviour and environment are stated. To achieve these changes, theory-based methods and practical strategies have to be selected in a third step. After this, step four is the creation of a programme plan, which includes the overall structure and themes of implementation as well as the development and testing of programme materials. The fifth step consists of the actual adoption and implementation of the guideline, which is evaluated in the sixth step [17].

A broad range of guideline implementation strategies with different effects has been described [87]. According to Grol and Grimshaw [10], “none of the approaches is superior for all changes in all situations; we probably need them all”. It is concluded that no single type of intervention is likely to be successful and, therefore, implementation efforts should use a combined approach of strategies tailored to the setting [46].

Evidence indicates that multifaceted implementation strategies are most effective [80,91,92], although Grimshaw et al. [93] argued that multifaceted strategies are not necessarily more effective than single interventions. The current recommendation to promote guideline adherence is to support guidelines by active and multifaceted implementation strategies [9], although the relative efficacy of each component within the multifaceted approach still remains unclear [14,43]. In addition to active implementation strategies, passive dissemination of guidelines should not be disregarded because it offers a cheaper and more feasible approach that may still be effective [94].

Although active implementation strategies have been effective in changing health professionals’ knowledge and practice, some studies have reported only moderate effects [71]. For example, absolute risk differences of 6% (95% CI: 1.8–15.9) for increasing use of endorsed professional practices with a continuing education intervention [95] and 12% (95% CI: 6.0–14.5) with the use of local opinion leaders [96] were reported in Cochrane systematic reviews. Nevertheless, it must be mentioned that the effectiveness in changing health professionals’ knowledge and practice varies both within and between studies. The results of these reviews are based on heterogeneous studies because the types of intervention, setting and outcomes differ [89,96]. Therefore, the setting and barriers need to be considered. Because guideline implementation and adherence do not happen immediately, but may take several years to complete, special attention must be given to the design of evaluation studies in the future [23]. This emphasizes the need for further research that will facilitate reliable and valid conclusions regarding the effectiveness of guideline implementation strategies [31].

### Limitations

This scoping review used a systematic approach to identify articles dealing with barriers and/or strategies for guideline implementation. It was conducted only in PubMed by using a comparatively narrow search algorithm. A broader search may lead to the identification of further articles. Nevertheless, we aimed to extract the barriers and strategies described in these articles to provide an overview of the topic.

Another major advantage of the process of summarizing suitable strategies for guideline implementation lies in the fact that there is only a limited evidence base describing the effects of single interventions, strategies or multifaceted approaches. Therefore, the attribution of strategies to barriers is based on aspects described or considered in the literature, although not all aspects are as yet based on the results of empirical research.

Furthermore, we excluded articles and aspects that are based on guideline- or disease-specific factors and focused on barriers and strategies that are more generalizable. Nevertheless, it is clear that, in the process of developing and implementing a guideline, the specific circumstances must be considered.

## 5. Conclusions

The development and implementation of guidelines is intended to improve the quality of care and to promote patient safety, by presenting the current evidence base and translating it into clinical practice. The publication and dissemination of guidelines does not, on its own, automatically result in their use. Therefore, some kind of implementation is needed. Until now, evidence on the effectiveness of different implementation strategies has been sparse. Nevertheless, the existing evidence indicates that a structured implementation can improve adherence to guidelines [10]. The barriers to guideline implementation and adherence in any particular case need to be analysed in advance so that strategies that are tailored to the specific setting and target groups can be developed. The strategy should include different types of interventions, and it should address physicians’ knowledge and attitudes in order to be effective in changing their behaviour. In considering these aspects, stakeholders must be included in order to reveal barriers and to develop adequate strategies for guideline implementation.

## Figures and Tables

**Table 1:** Studies included in qualitative synthesis.

Reference	Article Type	Location	Disease-Specific/Generic
[26]	Study	Germany	Disease-specific: Breast cancer
[27]	Study	USA	Disease-specific: Sinusitis and pharyngitis
[28]	Study	Scotland	Disease-specific: Dental care
[24]	Review	n/a	Generic
[29]	Study	England	Disease-specific: Depression
[30]	Review	n/a	Disease-specific: Stroke
[31]	Study	Netherlands	Disease-specific: Low back pain
[32]	Study	USA	Disease-specific: Thrombosis
[33]	Review	n/a	Generic
[22]	Review	n/a	Generic
[33]	Review	n/a	Disease-specific: Depression
[34]	Study	England, Scotland, USA	Generic
[35]	Study	Netherlands	Disease-specific: Diabetes
[36]	Study	Australia	Generic
[11]	Study	Australia	Disease-specific: Asthma
[37]	Study	Australia	Disease-specific: Asthma
[38]	Study	Australia	Disease-specific: Asthma
[39]	Review	USA	Disease-specific: Coronary care
[40]	Study	Denmark	Disease-specific: Peri-operative safety
[41]	Study	Netherlands	Disease-specific: Low back pain
[42]	Study	USA	Generic
[13]	Review	England	Generic
[23]	Study	Sweden	Disease-specific: Psychiatry
[43]	Review	n/a	Generic
[15]	Review	n/a	Generic
[44]	Study	USA	Disease-specific: Leukemia
[45]	Study	Germany, England, France, Spain, Italy, Poland	Disease-specific: Cardiovascular diseases
[46]	Study	USA	Disease-specific: Hypertension
[47]	Study	Netherlands	Disease-specific: Gynaecology
[48]	Review	USA, Australia, Netherlands, United Kingdom	Generic
[49]	Review	n/a	Disease-specific: Hypertension
[50]	Study	Australia	Disease-specific: Vascular diseases
[51]	Study	USA	Generic
[52]	Study	USA	Generic
[53]	Study	USA	Disease-specific: Angina
[54]	Review	Germany	Generic
[55]	Study	Spain	Disease-specific: Hypertension
[20]	Study	Netherlands	Generic
[56]	Study	Netherlands	Disease-specific: Urinary tract infection
[57]	Study	Netherlands	Disease-specific: Gynaecology
[58]	Study	USA	Generic
[59]	Study	New Zealand	Generic
[60]	Review	n/a	Generic
[61]	Study	Netherlands	Disease-specific: Gynaecology
[62]	Review	Germany	Generic
[63]	Study	Netherlands	Disease-specific: Anaemia
[64]	Review	Germany	Generic
[65]	Study	USA	Disease-specific: COPD
[66]	Study	Italy	Disease-specific: Diabetes
[67]	Study	Italy	Disease-specific: Diabetes
[68]	Review	Germany	Disease-specific: Heart failure
[69]	Review	n/a	Generic
[70]	Study	Netherlands	Disease-specific: Diabetes
[71]	Study	Australia	Disease-specific: Whiplash
[8]	Study	England	Disease-specific: Peri-operative fasting
[72]	Study	Canada	Disease-specific: Stroke
[73]	Study	Netherlands	Disease-specific: Anxiety and depression
[74]	Review	n/a	Disease-specific: Low back pain
[75]	Study	Estonia	Generic
[76]	Study	United Kingdom	Disease-specific: Tube feeding
[77]	Review	n/a	Generic
[78]	Review	n/a	Disease-specific: Cardiovascular diseases
[79]	Study	Netherlands	Disease-specific: Gynaecology
[80]	Review	n/a	Disease-specific: Physiotherapy
[81]	Study	Netherlands	Disease-specific: Anxiety
[82]	Study	Denmark	Disease-specific: Dementia
[83]	Review	n/a	Disease-specific: Oral health
[17]	Study	Netherlands	Disease-specific: Depression
[84]	Study	Netherlands	Disease-specific: Depression

n/a: not applicable.

**Table 2:** Barriers, interventions and strategies in guideline implementation.

Level	Barriers		Interventions	Strategies
**Personal factors** (related to physicians’ knowledge and attitudes)	Physicians’ knowledge	Lack of awareness	Increased dissemination of guideline	Dissemination strategies Standard dissemination (e.g., receiving guideline via e-Mail) Dissemination of training material Continuing education Active learning from experts: opinion leaders Educational meetings Individualized feedback and group performance audit Quality circle
			Use of mass media to increase awareness	
			CME	
		Lack of familiarity	Making guideline available with practical instruments	
			Educational posters in examination rooms	
			CME that focuses on specific guideline recommendations	
	Physicians’ attitudes	Lack of agreement	Opinion leaders	Educational meetings Educational outreach visits Marketing outreach visits Identifying opinion leaders Financial opportunities/penalties Standing orders
			Physician participation in guideline development	
			Special society endorsement of guideline	
			Small group education	
		Lack of self-efficacy	CME focusing on skills	Dissemination Educational outreach visits (individualized) audit and feedback
			Interactive learning / group training	
			Audit and feedback of individual performance: positive individualized feedback during training and subsequently in practice, assistance with questions	
		Lack of skills	CME focusing on skills	
			Audit and feedback of individual performance	
		Lack of learning culture	Promoting learning organizations	
		Lack of outcome expectancy	Audit and feedback of practice wide performances	
			Citation of previous published success at improving outcomes through guideline implementation	
		Lack of motivation	Motivational strategies that utilize audit and feedback	
			Opinion leaders	
**Guideline- related factors**	Lack of evidence		Use of methods of evidence-based medicine	Use of methods of evidence-based medicine for guideline development Communication strategies Marketing outreach visits (Computerized) decision support systems Reminders Pilot projects
			Appraisal of evidence in recommendations	
			Regular updates	
	Plausibility of recommendations		Short and user-friendly versions of guidelines	
			Checklists	
	Complexity (too theoretical)		Simplicity Design and development of guideline	
	Poor layout			
	Access to guideline		Provide easy access to guideline	
			Decision support systems	
	Lack of applicability		Using tablets, smartphones, and mobiles for provision of guidelines	
	Focus on patients with single disease entities		Consideration of comorbidity and multimorbidity in guidelines	
	Exclusion of patients with complex disease entities			
	Lack of clear intervention goals		Setting clear intervention goals	
	Trialability		Pilot projects	
**External factors**	Organisational constraints		Standardisation of processes and procedures	Improvements in organisation of care
			Development of protocols specifically targeting practice assistants	
			Guideline development needs to consider the care setting	
			Link to quality management	
	Lack of resources (time restrictions, heavy workload, facilitation)		Financial incentives/compensation	Standing orders
			Providing time for documentation and utilization of guidelines	
			Clear roles	
			External facilitation	
	Lack of collaboration		Improving multiprofessional collaboration with other healthcare professionals	Local adaptation Local consensus groups Incorporation into established structures
	Social and clinical norms		Local consensus groups	

CME: Continuous Medical Education.

## Abbreviations

The following abbreviations are used in this manuscript:

CI	Confidence Interval
CME	Continuous Medical Education
PRISMA	Preferred Reporting Items for Systematic Reviews and Meta-Analyses
USA	United States of America
